# Automated production of *CCR5*-negative CD4^+^-T cells in a GMP-compatible, clinical scale for treatment of HIV-positive patients

**DOI:** 10.1038/s41434-021-00259-5

**Published:** 2021-04-19

**Authors:** Lea Isabell Schwarze, Tanja Sonntag, Stefan Wild, Sabrina Schmitz, Almut Uhde, Boris Fehse

**Affiliations:** 1 Research Department Cell and Gene Therapy, University Medical Centre Hamburg-Eppendorf, Hamburg, Germany; 2 German Centre for Infection Research (DZIF), partner site, Hamburg, Germany; 3 Miltenyi Biotec, Bergisch Gladbach, Germany

**Keywords:** Immunotherapy, Infectious diseases

## Abstract

Ex-vivo gene editing in T lymphocytes paves the way for novel concepts of immunotherapy. One of those strategies is directed at the protection of CD4^+^-T helper cells from HIV infection in HIV-positive individuals. To this end, we have developed and optimised a *CCR5*-targeting TALE nuclease, CCR5-Uco-hetTALEN, mediating high-efficiency knockout of C-C motif chemokine receptor 5 (CCR5), the HIV co-receptor essential during initial infection. Clinical translation of the knockout approach requires up-scaling of the manufacturing process to clinically relevant cell numbers in accordance with good manufacturing practice (GMP). Here we present a GMP-compatible mRNA electroporation protocol for the automated production of *CCR5*-edited CD4^+^-T cells in the closed CliniMACS Prodigy system. The automated process reliably produced high amounts of *CCR5*-edited CD4^+^-T cells (>1.5 × 10^9^ cells with >60% *CCR5* editing) within 12 days. Of note, about 40% of total large-scale produced cells showed a biallelic *CCR5* editing, and between 25 and 42% of produced cells had a central memory T-cell phenotype. In conclusion, transfection of primary T cells with CCR5-Uco-hetTALEN mRNA is readily scalable for GMP-compatible production and hence suitable for application in HIV gene therapy.

## Introduction

In the last years, genome editing has rapidly turned from a niche technology to a broadly used approach in basic and applied science. The potential of targeted genome modification using designer nucleases for human gene therapy has been already recognised for zinc-finger nucleases (ZFNs). In fact, the very first human gene therapy application of genome editing was based on ZFN-mediated knockout of the C-C motif chemokine receptor 5 [CCR5] [1]. CCR5 came into focus, since, besides its physiological function, it serves as an HIV co-receptor on the surface of CD4^+^-T cells and macrophages [2, 3]. Indeed, the essential role of CCR5 for successful HIV infection was established by the identification of a homozygous, naturally occurring 32 bp deletion in the open reading frame of *CCR5* (*CCR5*Δ32) in multiply-exposed, but non-infected individuals [3, 4]. Two observations have made CCR5 a favourite target for HIV gene therapy: First, the above mentioned almost complete protection of homozygous individuals [4, 5] from HIV. Second, reported cases of obviously complete virus elimination in individuals with an established HIV infection after haematopoietic stem cell transplantation from *CCR5*Δ32-homozygous donors [6, 7].

Even though anti-retroviral therapy (ART) has turned AIDS from a deadly into a chronic, largely manageable disease with nearly normal life expectancy, gene therapy is still an attractive treatment option. Importantly, ART does not eliminate the HI virus. Moreover, although ART suppresses HIV replication below detectable levels, it is not able to fully prevent chronic immune activation, inflammation and slow lymphoid tissue damage [8–10]. Finally, lifelong ART increases the risk of drug- resistance development and typical problems of continuous therapies such as accumulating toxicity, adverse drug interaction and decreasing compliance [11].

Previously, a variety of gene-therapy approaches were investigated, targeting different steps of the HIV life cycle. Whereas early efforts were mainly directed at “intracellular immunisation” [12], entry inhibition came into focus right thereafter [13]; clinical studies addressed both CD4^+^-T cells [14, 15] and CD34 haematopoietic stem cells (HSC) [16, 17]. More recently, different strategies of genome editing were tested in preclinical settings. These included blockade of HIV entry via *CCR5* knockout using different types of designer nucleases, namely ZFN [18], TAL effector nucleases [TALEN] [19] and CRISPR/Cas9 [20], but also HIV provirus excision using designer recombinases [21] as well as nucleases [22].

We earlier reported development of a highly active TAL effector nuclease, CCR5-Uco-TALEN, targeting the *CCR5* gene [23]. We also showed that mRNA electroporation represents a promising approach for the transient expression of TALENs [24]. In an accompanying work, we present data on the further improved CCR5-Uco-hetTALEN with high translation potential [25]. However, for clinical application of any (immuno-)therapy based on ex-vivo genome-edited T cells, methods for large-scale production in accordance with good manufacturing practice (GMP) will be required. We here describe the development of an mRNA-electroporation based GMP-compatible large-scale protocol for the generation of *CCR5*-edited T cells in the CliniMACS Prodigy. We show that the automated process facilitates production of clinically relevant numbers of gene-edited CD4^+^-T cells with high rates of biallelic *CCR5* gene editing. Moreover, we characterise the large-scale produced *CCR5*-edited CD4^+^-T cells in view of their potential clinical applicability.

## Material and methods

### Next-generation amplicon sequencing

Potential off-targets were calculated in silico using the online bioinformatic tools PROGNOS [26] and the Paired Target Finder from TAL Effector Nucleotide Targeter 2.0 [27]. Applied search parameters and description of amplicon next-generation sequencing (NGS) by Microsynth AG (Balgach, Switzerland) are summarised in Schwarze et al [25]. Analysed targets are shortly described in Table S1. All reads containing insertions and deletions (Indels) at and between TALEN binding sites (“target region”) were considered editing-induced. Reads without Indels at the target region site were counted as wildtype (WT). Statistical analysis of Indel ratio was done using a one-tailed Welch’s *t*-test due to unequal sample size and variance. All other graphs, as well as statistical analyses were performed using GraphPad Prism 8.4.3.

### mRNA production

CCR5-Uco-hetTALEN L + R mRNA was produced by in-vitro transcription from T7- and poly(A)-plasmids containing CCR5-Uco-hetTALEN L or R [25] in large-scale by contract manufacturer BioNTech IMFS (Idar-Oberstein, Germany) in a GMP-like protocol. A 5’ ARCA cap was added during in-vitro transcription of the RNA, and mRNA was purified using silica beads.

### DNA and RNA isolation

Genomic DNA (gDNA) from sampled cells was isolated using the QIAamp DNA Blood Mini Kit (QIAGEN, Hilden, Germany) according to the manufacturer’s protocol. Concentration of isolated gDNA was assessed using the Qubit 2.0 Fluorometer together with the Qubit dsDNA BR Assay Kit (ThermoFisher Scientific, Waltham, MA, USA) following the manufacturer. The gDNA from samples of restimulated cells was isolated using the QIAamp DNA Micro Kit (QIAGEN). The concentration of those samples was measured using the DS11 Plus (DeNovix, Wilmington, DE, USA). Total RNA was extracted from sampled cells using the RNeasy Mini Kit (QIAGEN) according to the manufacturer’s spin protocol for animal cells. Homogenisation of cells was performed using QIAshredder spin columns from QIAGEN. The iScript Advanced cDNA Synthesis Kit for RT-qPCR (Bio-Rad, Hercules, CA, USA) was used for reverse transcription. For each cDNA synthesis 15 µl total RNA (≤7.5 µg) were used as template for qPCR. The RNA concentration was determined with a Qubit 2.0 Fluorometer using the Qubit RNA HS Assay Kit (ThermoFisher Scientific) in accord with the manufacturer’s protocol.

### Droplet digital PCR (ddPCR)

Droplet digital PCR was performed using the Bio-Rad QX100 system according to manufacturer’s protocols. QuantaSoft 1.7.4.0917 (Bio-Rad, Hercules, CA, USA) was used to analyse data from ddPCR. A detailed protocol for gene editing frequency ddPCR (GEF-dPCR) performance and analysis has been published elsewhere [28]. Primers and probes for ddPCR assays are listed in Table S2. The assay for *CCR5* gene editing rates was performed with CCR5fw, CCR5rv, CCR5ref and CCR5mut primers and probes. *CCR2* gene editing rates were determined using CCR2fw, CCR2rv, CCR5ref and CCR2mut primers and probes. Frequencies of large deletions at the on-target *CCR5* and off-target *CCR2* were assessed with dPCR using hEPORfw, hEPORrv and hEPORref primers and probes, as well as the following primers and probes for the individual targeted assays: (dKO) CCR2fw, CCR5rv, CCR5ref; (inversion) Inv1fw, CCR2rv, CCR5ref; (integration) Int1fw, CCR2rv.

### Real-time qPCR

All primers used in real-time qPCRs (RT-PCRs) and melting-curve analyses are depicted in Table S2. Diluted CCR5-Uco-hetTALEN L + R plasmids were used to create standard curves for calculation of copy numbers (Fig. S1). All RT-PCRs were done in triplicates.

Quantification of CCR5-Uco-hetTALEN copy numbers in RNA isolates was performed with 2 µl template using the TB Green *Premix Ex Taq* (Tli RNaseH Plus) Kit from Takara Bio (Mountain View, CA, USA) according to the manufacturer’s protocol for the LightCycler 480 Instrument II (LC480 System). CCR5-Uco-hetTALEN was detected after reverse transcription using primers hetTALENfw and hetTALENrv (detects both mRNA and plasmid) or primers Kanfw and Kanrv (detects plasmid, only) at final concentrations of 0.4 µM. Importin 8 (IPO8) was used as an external reference gene with the IPO8fw and IPO8rv primer pair at a final concentration of 0.4 µM. Crossing point (C_P_) calculations by LC480 software (version 1.5) for absolute quantification analysis was performed using the second derivative maximum method or using the fit points method, if plasmid was detected. Plasmid copy numbers were calculated, if at least two of the three replicates showed correct melting temperature of >89.0 °C.

Detection of CCR5-Uco-hetTALEN plasmid in gDNA isolates was performed with at least 20 ng template using the Maxima SYBR Green/Rox qPCR Master Mix (ThermoFisher Scientific) according to manufacturer’s (two-step) protocol at the LC480. Crossing point (C_P_) calculations by LC480 software (version 1.5) for absolute quantification analysis were performed using the fit points method.

### Single-cell high-resolution melting curve analysis (scHRMCA)

After sorting of single cells treated with CCR5-Uco-hetTALEN (or non-treated cells as control) into a 96-well PCR plate containing 10 µl of lysis buffer [29], the plate was incubated at 37 °C for 1 h and at 95 °C for 10 min. First PCR was performed using the following primers: nesPCRfw and nesPCRrv. The resulting PCR product was diluted 1:80 with dH_2_O and used as template for melting-curve analysis at the LC480 with primers HRMfw and HRMrv. Non-edited single cells were used as control to compare melting profiles. A more detailed protocol can be found in Schwarze et al [25].

### Production of lentiviral particles

Gibbon-ape-leukaemia-virus-envelop (GALVenv) pseudotyped LeGO-S vectors encoding T-Sapphire and CCR5-tropic HIVenv (BaL-env) lentiviral particles encoding mCherry (LeGO-C) [30] were produced as previously described by Mock et al [24, 31]. Viral supernatants were titrated on PM1 cells in the presence of 8 µg/ml DEAE-dextran [30].

### Restimulation of frozen *CCR5*-edited cells

To test the capacity of large-scale produced *CCR5*-edited CD4^+^-T cells after freezing, cells were thawed and restimulated with anti-CD3/anti-CD28 beads (T Cell TransAct, human from Miltenyi Biotec, Bergisch-Gladbach, Germany) at a concentration of 1:500 in supplemented TexMACS Medium from Miltenyi Biotec (+3% human serum (H6914, Sigma-Aldrich, St-Louis, MO, USA), 625 U/ml Human IL-7, research grade and 87.5 U/ml Human IL-15, research grade (both from Miltenyi Biotec).

### Infection assay

For this assay, 1 × 10^5^ *CCR5*-edited cells from all runs (#1-#4) were seeded in 250 µl supplemented TexMACS Medium (3% human serum (H6914, Sigma-Aldrich), 625 U/ml Human IL-7, research grade and 87.5 U/ml Human IL-15, research grade (both Miltenyi Biotec)) with 8 µg/ml DEAE-dextran in triplicates into a 48-well culture plate 3 days after restimulation. After addition of viral vector supernatants LeGO-S_GALVenv and LeGO-C H_IVenv to each well, cells were centrifuged at 1000 x *g* for 1 h at room temperature. Gene transfer rates were measured 4 days post transduction at the BD LSRFortessa flow cytometer using the following laser/filter combinations: mCherry = 561 nm laser, filters 600 and 610/20; T-Sapphire = 405 nm laser, filters 475 and 525/50.

### Proliferation assay

Proliferation of *CCR5*-edited cells produced at the CliniMACS Prodigy was monitored using CellTrace CFSE Cell Proliferation Kit from Invitrogen (Carlsbad, CA, USA). One day after thawing and restimulation with T Cell TransAct human 1:500 (Miltenyi Biotec), the cells were stained with 0.5 µM CellTrace CFSE dye according to the manufacturer’s instruction. CSFE fluorescence was daily measured by flow cytometry at the BD LSRFortessa for 7 days. Prior to each measurement, cells were additionally stained with 7AAD (Biolegend, San Diego, CA, USA) and anti-CCR5-PE (REA245, Miltenyi Biotec). Cell staining was performed in 50 µl CliniMACS PBS/EDTA Buffer supplemented with 0.5% bovine serum albumin (Miltenyi Biotec) with 2.5 µl of 7AAD and 1 µl CCR5-PE-antibody for 10 min in the dark at room temperature. The following laser/filter combinations were used for the measurements: CSFE = 488 nm laser, filters 505 and 530/30; 7AAD = 561 nm laser, filters 635 and 670/30; PE = 561 nm laser, filters 575 and 582/15.

### Cytokine detection assay

Detection of cytokines (GM-CSF, IFN-α, IFN-γ, IL-2, IL-4, IL-5, IL-6, IL-9, IL-10, IL-12p70, IL-17A and TNF-α) was performed using the MACSPlex Cytokine 12 Kit from Miltenyi Biotec following the manufacturer’s protocol. Cell acquisition was performed at the MACSQuant Analyzer 10 Flow Cytometer (Miltenyi Biotec) using the Express Modes MACSPlex_Standard and MACSPlex_Sample. Calibration of the MACSQuant 10 was performed prior to measurement with MACSQuant Calibration Beads. Flow-cytometry results were analysed using MACSQuantify 2.13.1 software. Undiluted medium (harvested on day 9 after restimulation) from runs #1-#4 was used in duplicates for this assay. Medium from non-edited CD4^+^-T cells from small-scale experiments harvested on day 12 after first stimulation was used as a control in duplicates. Data represents average results of three different donors. The detailed protocol for small-scale cell cultivation and the obtained data are described in Schwarze et al [25].

### Automated T-Cell engineering (TCE) at CliniMACS prodigy

Automated TCE was performed at the CliniMACS Prodigy using the T Cell Engineering Process (beta-version V0.7.0.1). If not stated otherwise, all materials and reagents were purchased from Miltenyi Biotec. TCE was performed with the CliniMACS Prodigy Tubing Set TS 520 connected to the CliniMACS Prodigy EP-2. The tubing sets were connected via sterile welding using the TSCD-II (Terumo BCT inc. Lakewood, CO, USA). Integrity tests and priming of tubing sets with CliniMACS PBS/EDTA Buffer supplemented with 0.5% human serum albumin (Kedrion Biopharma, Barga, Italy) connected over valve 1 and supplemented TexMACS GMP Medium were performed according to manufacturer’s instructions. The TexMACS GMP Medium supplemented with 3% human serum (H6914, Sigma-Aldrich), 12.5 ng/ml MACS GMP Recombinant Human IL-7 and 12.5 ng/ml MACS GMP Recombinant Human IL-15 was connected via sterile welding to valve 3 and covered with an opaque foil. Fresh buffy coats from healthy donors were used as starting material for CD4^+^ cells separation in the CliniMACS Prodigy. All buffy coats used in these experiments were kindly provided by the Institute of Transfusion Medicine at the UKE; they represented leftovers from erythrocyte concentrate production from whole-blood donations by healthy blood donors, released for research after informed consent. The donor material was tested for cell count, number of CD4^+^-T cells, and immune cell composition before sterile welding to the tubing set over the application bag. CD4^+^ cells separation was performed using CliniMACS CD4 Reagent connected to valve 2. The cells were labelled with an incubation time of 15 min at 4 °C (‘1 vial option’ at installation start). After separation, cells were again tested for cell count and purity. Culture was started immediately with 2.0 × 10^8^ cells (if applicable) in 70 ml supplemented TexMACS GMP Medium at 37 °C and 5% CO_2_. The MACS GMP T Cell TransAct was connected via valve 4 and completely added to cells for activation. The activity matrix at the CliniMACS Prodigy was set according to Table 2. Shaker type 2 was activated at culture start and deactivated on day 3 before electroporation, except for run #2. 30 ml medium was added to the cell culture on day 1. The electroporation was performed on day 3. To do so, cells were automatically washed and rebuffered in 20 ml CliniMACS Electroporation Buffer, which was connected via sterile welding. The indicated amounts (Table 1) of CCR5-Uco-hetTALEN L + R mRNA (produced in-vitro by BioNTech IMFS) were diluted in 5.2 ml of CliniMACS Electroporation Buffer and injected over a sterile filter into the primed nucleic acid bag. Further electroporation parameters are described in Table 3. After each electroporation step, cells were transferred back into the culture chamber inside the CentriCult-Unit containing 66 ml supplemented TexMACS GMP Medium. The electroporated cells were kept at 32 °C for 24 h without shaker. About 17 h after electroporation start, 1 cycle of culture wash was performed to remove CliniMACS Electroporation Buffer. Shaker type 2/3 was activated on day 4/5. Media exchanges and culture washes were performed automatically by the device once each day. If required, the ‘media and waste bag’ was exchanged once during the process following the device instructions. Samples were taken prior to electroporation and on days 4, 5, 6, and 12 via the attached QC pouches following the device instructions. The harvest of cells was performed on day 12 choosing harvest type 1. Cells were either harvested in media (run #1) or in 0.9% NaCl solution for infusion from Fresensius Kabi (Bad Homburg, Germany). After the harvest, cells were either collected for further experiments or frozen in human sera containing 10% Dimethylsulfoxid, DMSO (both Sigma-Aldrich) in liquid nitrogen.

**Table 1:** Cell numbers and mRNA amounts used in TCE runs.

Run	Starting cell number	mRNA
#1	2 × 10^8^ cells	720 µg per arm
#2	2 × 10^8^ cells	600 µg per arm
#3	2 × 10^8^ cells	600 µg per arm
#4	1.4 × 10^8^ cells	240 µg per arm

### Flow cytometry at the MACSQuant

All antibodies used in the panels were conjugated, anti-human antibodies purchased from Miltenyi Biotec. The immune-cell composition and purity were determined using the following antibodies: CD45-Vioblue (clone REA747), CD3-FITC (REA613), CD4-Viogreen (REA623), CD16-PE (REA423), CD56-PE (REA196), CD14-APC (REA599), CD19-PEVio770 (REA675), CD8-APCVio770 (REA734). T-cell stemness was analysed using the following panel: CD197-Vioblue (REA546), CD4-Viogreen (REA623), CD3-FITC (REA613), CD95-PE (REA738), CD62L-APC (145/15), CD45R0-PEVio770 (UCHL1), CD8-APCVio770. Expression of CCR5 was measured during the process using the following panel: CD45-Vioblue (clone REA747), CD3-FITC (REA613), CD4-Viogreen, CCR5-PE (REA245), CCR2-APC (REA264), CD8-APCVio770 (REA734). T-cell exhaustion was measured using the following antibody panel: CD223-Vioblue (REA351), CD4-Viogreen (REA623), CD3-FITC (REA613), CCR5-PE (REA245), CD279-PEVio770 (PD1.3.1.3), CD366-APC (REA635), CD8-APCVio770 (REA734). Cell viability was determined by 7AAD (Miltenyi Biotec) staining. The staining of cells was performed in DPBS (Gibco, ThermoFisher Scientific) supplemented with 0.5% bovine serum albumin, 2 mM EDTA Dihydrate (both Sigma-Aldrich) and antibodies at a dilution of 1:50. 10 µl of 7AAD solution were added freshly to each panel mix. The cells were stained for 10 min at room temperature in the dark. Red blood cell lysis was performed after staining of cells from buffy coat prior to separation. Cell acquisition was performed at the MACSQuant Analyzer 10 Flow Cytometer (Miltenyi Biotec). Calibration of MACSQuant 10 was performed weekly with MACSQuant Calibration Beads. Compensation of multicolour panels was performed using MACS Comp Bead Kit, anti-REA (Miltenyi Biotec). Flow-cytometry results were analysed using FlowJo v10.6.2 or MACSQuantify 2.13.1.

## Results

### Outline of the process

**Fig. 1:**
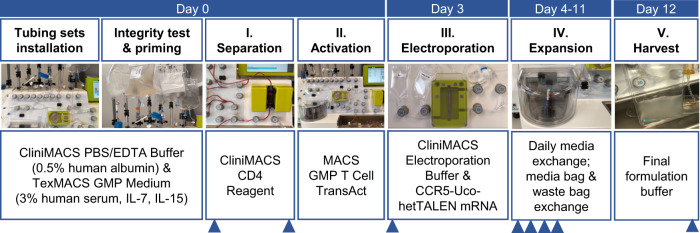
Workflow of the automated T Cell Engineering (TCE) process and set up of the tubing sets at the CliniMACS Prodigy. *CCR5*-edited CD4^+^-T cells are manufactured during a 12-day process after single installation of a fused tubing set. Day 0: Installation of tubing sets (TS 520 + EP-2), integrity test and priming after attachment of supplemented CliniMACS PBS/EDTA (0.5 % human albumin) and TexMACS GMP Medium (MACS GMP Recombinant Human IL-7 and IL-15 and 3% human serum). CD4^+^ cells were separated after positive magnetic labelling with CliniMACS CD4 Reagent from fresh buffy coats and subsequently activated using MACS GMP T cell TransAct. Day 3: Automated electroporation of cells in CliniMACS Electroporation Buffer with TALEN mRNA and incubation of cells at 32 °C for 24 h. Day 4–11: Expansion phase with feeding or media replacing steps with supplemented TexMACS GMP Medium. Medium and waste bags were exchanged during these days. Day 12: Harvest of cells in final formulation buffer. During the whole process, the cell culture was regularly agitated to ensure sufficient gas support. Blue triangles indicate time points of sample collection on day 0 before and after activation, on day 3 prior to electroporation, on days 4–7 and after harvest on day 12.

To develop and assess a GMP-compatible, automated protocol for the production of *CCR5*-edited CD4^+^-T cells by TALEN-mRNA electroporation, we carried out four independent runs (#1-#4) on the CliniMACS Prodigy. All runs were performed using the CliniMACS Prodigy Tubing Set TS 520 connected to the CliniMACS Prodigy EP-2, which was installed at the CliniMACS Prodigy. Each individual run included the following steps after tubing set installation: (i) separation of CD4^+^ cells from buffy coats of healthy donors and activation of T cells, (ii) electroporation with CCR5-Uco-hetTALEN L + R mRNA, (iii) expansion of T cells and (iv) harvest of the cell product. During all runs, samples were taken for quality controls on days 0, 3–7 and 12 to monitor quality and quantity of cells (Fig. 1). Monitoring included phenotypic analysis, determination of gene-editing rates and CCR5-Uco-hetTALEN decay measurements.

### CD4 enrichment and activation

**Fig. 2:**
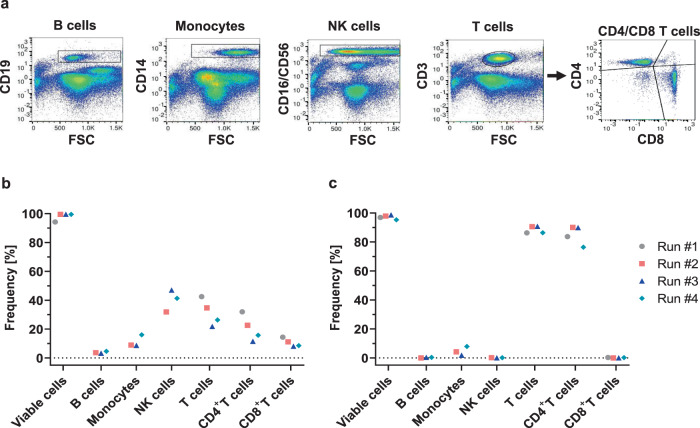
The automated TCE process facilitates efficient separation of vital CD4^+^ cells. **a** Exemplary immune-cell composition of starting material (buffy coat) as established by flow cytometry for run 2. All plots show CD45^+^7AAD^−^ cells. **b** Summarised data on immune cell composition and viability of cells from buffy coat before separation for all four runs (for the first run, B-cell and monocyte data was not available). **c** Summarised data on immune cell composition and viability and after separation for all four runs (for the first run, B-cell and monocyte data was not available).

**Fig. 3:**
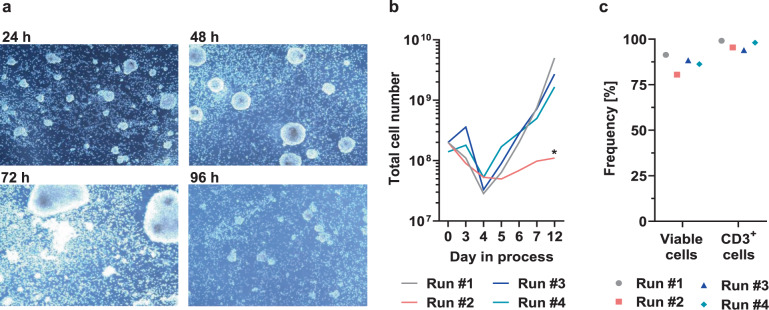
The automated TCE process facilitates efficient expansion of vital T cells. **a** Pictures from culture 2 taken with the internal camera of the CliniMACS Prodigy 24 h, 48 h, 72 h and 96 h after activation under static culture conditions. Picture at 72 h was taken before electroporation. **b** Calculated total cell numbers in TCE process for each run on days 0, 3, 4, 5, 6, 7, and 12. **c** Viability of cells measured by flow cytometry based on staining with 7AAD. Frequency of viable (7AAD-negative) cells and viable CD3^+^ cells on day 12 after final harvest. *Please note that on day 2 of run #2 a 9 h interruption occurred in the central CO_2_ supply chain of our research building.

CD4 cell enrichment was performed using the CliniMACS CD4 Reagent. The buffy coat and separated cells were analysed for immune cell composition by flow cytometry. Cells were stained with an antibody panel for detection of B cells, monocytes, NK as well as CD4^+^- and CD8^+^-T cells. Populations were gated and assigned as one of the immune cell types as shown in Fig. 2a. Total amounts of CD4^+^-T cells ranged from around 15 to 32% in the initial samples from buffy coats (Fig. 2b) and reached 76 to 90% in the enriched products (Fig. 2c). We observed some co-enrichment of monocytes, most probably due to their low CD4 expression, whereas B and NK cells were effectively removed from the target cell population after separation (Fig. 2c). Cell viability was defined based on 7AAD negativity; average viabilities were 98.2 ± 2.3% for starting preparations and 97.2 ± 1.3% after enrichment (Fig. 2b, c). Cultures were started with total numbers of 2 × 10^8^ cells, except for one run (#4), where lower cell numbers had been obtained (Table 1). The activity matrix was programmed as outlined in Table 2. In order to visualise cell growth and appearance with the internal microscope camera, the shaker was deactivated from day 0 to day 4 during run #2. Upon activation, T cells formed typical clusters, which grew over time (pictures 24–72 h). Cell clusters were disrupted after electroporation as seen at the photograph taken 96 h after activation (Fig. 3a).

**Table 2:** Final protocol for activity matrix of TCE process.

Day	Activity	Details
0	Activate shaker	Type 2
1	Feed Medium	30 ml
3	Electroporation Set temperature	Ep.v. 20 ml, r.v. 66 ml 32 °C
4	Culture wash Activate shaker Set temperature	1 cycle Type 2 37 °C
5	Media exchange Activate shaker	−125 ml/+175 ml Type 3
6	Media exchange	−125 ml/+125 ml
7	Culture wash; Feed Medium	1 cycle/+ 50 ml
8	Media exchange	−180 ml/+180 ml
9	Media exchange	−180 ml/+180 ml
10	Media exchange	−180 ml/+180 ml
11	Media exchange	−180 ml/+180 ml
12	Harvest	Type 1

*Ep.v.* electroporation volume, *r.v.* recovery volume in chamber.

### Electroporation and expansion

3 days after cell separation and activation, electroporation of CCR5-Uco-hetTALEN L + R mRNA was performed. For the first experiment, mRNA amounts for the large-scale run at the CliniMACS Prodigy were extrapolated based on titrations of purchased mRNAs in small-scale experiments (Fig. S2). In view of the observed high editing rates, mRNA amounts were decreased stepwise in the course of the four runs (Table 1). The indicated amounts of CCR5-Uco-hetTALEN L + R mRNA per arm were dissolved in CliniMACS Electroporation Buffer and transferred to the nucleic acid bag. Cells from culture chamber were automatically rebuffered in 20 ml CliniMACS Electroporation Buffer, filtered and transferred to bag 2 of CliniMACS Prodigy EP-2. Cells and dissolved mRNA were automatically mixed by passing through the cuvette and electroporated using the parameters depicted in Table 3 in multiple small portions until cell solution was empty. After each single round, electroporated cells were transferred back to the culture chamber. To enhance TALEN-mediated gene editing, cells were cultured at 32 °C for 24 h after electroporation [32]. Thereafter, i.e. 1 day post electroporation, cell numbers were found to be strongly reduced (Fig. 3b). However, at this time point actual cell numbers were difficult to determine due to the formation of large cell clumps after the washing step. Importantly, subsequent growth kinetics indicated no major impact of electroporation and hypothermic conditions on cell vitality and replication (Fig. 3b, c).

**Table 3:** Electroporation parameter used in TCE runs.

Pulse number	Parameter	Setting
1	Voltage	600 V
	Length	120 μs
	Mode	burst
	Direction	bipolar
	Burst duration	8 μs
2	Voltage	500 V
	Length	1000 μs
	Mode	burst
	Burst duration	8 μs

During the expansion phase, supplemented TexMACS GMP Medium was exchanged automatically every day as defined by the activity matrix (Table 2). Final products were harvested on day 12. Total cell numbers in the final products ranged from 1.65 × 10^9^ to 5.00 × 10^9^ cells for runs #1, #3 and #4 (Fig. 3b). CD3^+^ cells constituted at least 94% of the final products (Fig. 3c). For runs #1, #3 and #4, the mean cell viability was 88.7% ± 2.1. In contrast, only app. 1 × 10^8^ cells with a viability of app. 80% were harvested after run #2 (Fig. 3b, c). Importantly, central CO_2_ supply of our research building was interrupted for 9 h at the second day of run #2, i.e. during the T-cell activation phase. The interrupted gas supply obviously strongly impaired viability and growth potential of activated T cells.

### Phenotypic characterisation of large-scale produced cells

**Fig. 4:**
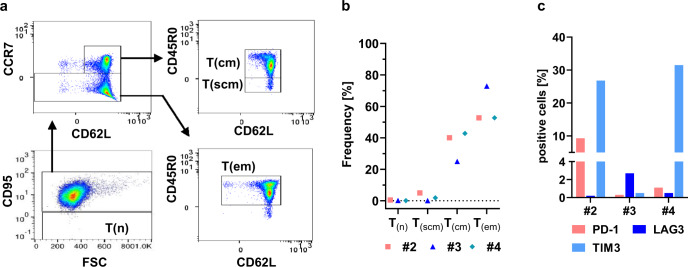
The automated TCE process facilitates efficient expansion of gene-edited central-memory T cells without showing exhaustion. **a** Characterisation of T-cell subsets based on expression of markers CD95, CCR7, CD62L and CD45R0. Exemplary flow cytometry plots for run #4 are shown. CD3^+^CD4^+^ viable cells gated for naïve T(n): CD95, stem cell memory T(scm): CD95+, CCR7+, CD62L+, CD45R0-, central memory T(cm): CD95+, CCR7+, CD62L+, CD45R0+ and effector memory T(em): CD95^+^, CCR7^-^, CD45RO^+^. **b** Comparison of frequency of T_(n)_, T_(scm)_, T_(cm)_ and T_(em)_ for runs #2-#4. Gating based on CD3^+^CD4^+^ viable cells. **c** Frequency of PD-1-, LAG3- and TIM3-positive viable CD3^+^CD4^+^ cells for runs #2-#4 measured on process day 12.

Ideally, edited T cells should be long-lived to ensure protection for extended time frames, but survival- and self-renewal rates are heterogeneous among different T-cell subpopulations. Naïve T cells can be distinguished from memory T cells, which can be further subdivided into stem cell memory, central memory and effector memory T cells. Self-renewal and survival are lowest in effector memory T cells and highest in stem cell memory T cells. T-cell subsets were characterised by flow cytometry for runs #2-#4. To this end, viable CD3^+^CD4^+^ cells were classified based on expression of CD95, CD62L, CCR7 and CD45R0 (Fig. 4a). T effector memory cells (T(em): CD95^+^, CCR7^-^, CD45RO^+^) were the most prevalent subset, whereas T central memory cells (T(cm): CD95^+^, CCR7^+^, CD45RO^+^), represented about 25–42% of all CD3^+^CD4^+^-T cells (Fig. 4b). As expected, no naïve T cells (T(n): CD95^-^) were present in the final product of all analysed runs. Expression of CD62L was heterogeneous in effector memory T cells. The proportion of stem cell memory T cells was diverse and ranged from 0 to 5% (Fig. 4b). Besides the T-cell subpopulations, CCR5 expression was also monitored on edited cells by flow cytometry on different days during runs #2-#4. On day 12 of the process, 11.4%, 5.6% and 15.9% of the CD4^+^-T cells were positive for CCR5 for runs #2-#4, respectively (Fig. S3). If compared to the highest measured number of CCR5-positive cells during each process these results correspond to reductions in CCR5 expression by 30.8% for run #2, 77.8% for run#3 and 54.2% for run #4. However, CCR5 expression is very heterogeneous and comparison to mock-treated cells of the same donor was not possible in the large-scale setting; consequently, these numbers likely underestimate the actual reduction of CCR5 expression in TALEN-treated cells.

Cultivation of activated T cells, as well as their electroporation with a designer nuclease might impair fitness of treated cells. Therefore, the *CCR5*-edited cells were examined for expression of exhaustion markers PD-1, LAG3 and TIM3 on living CD3^+^CD4^+^ cells by flow cytometry on day 12 of the process for runs #2-#4 (Fig. 4c). All analysed runs showed no sign of extended T-cell exhaustion, as would be indicated by high expression of all measured markers. We found minor fractions of cells positive for PD-1 (9.3%) and TIM3 (26.8%) in run #2, for run #4 about 31.5% of CD4^+^ T cells were positive for TIM3.

### *CCR5* on-target and *CCR2* off-target editing

**Fig. 5:**
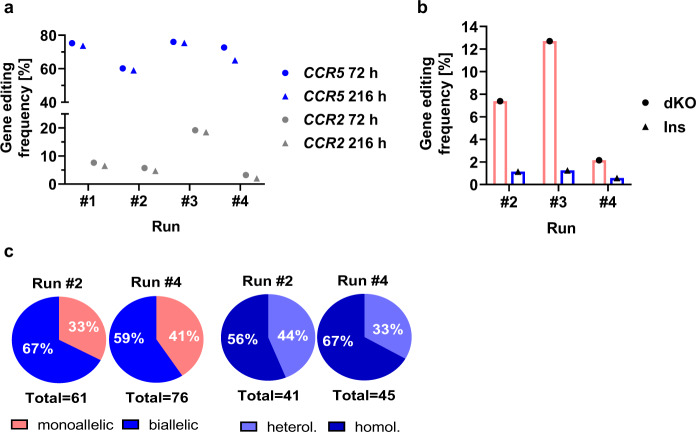
Characterisation of cells from TCE process by dPCR and scHRMCA. **a** Gene-editing rates for *CCR5* (blue) and *CCR2* (grey) calculated from GEF-dPCR data for all 4 TCE processes (runs #1-#4) at 72 h and 216 h post electroporation. **b** Deletion (dKO) and insertion (Ins) rates for the 15 kb fragment between CCR5-Uco-hetTALEN cut sites at *CCR5* and *CCR2* calculated from dPCR data for 3 TCE processes (runs #2-#4) at 216 h post electroporation. **c** scHRMCA for single cells from runs #2 and #4 based on monoallelic and biallelic, as well as heterologous (heterol.) and homologous (homol.) biallelic melting profiles.

To monitor gene editing rates, *CCR5* and *CCR2* Indel rates were determined by GEF-dPCR at two relevant points in the process—first at the earliest possible point after electroporation (72 h post electroporation), and at the day of final harvest (216 h post electroporation) to ensure successful gene editing. At the two time points very similar gene editing rates for each *CCR5* and *CCR2* were found, indicating identical growth kinetics of *CCR5*-edited and WT cells. Final products contained between 65% and 75% on-target *CCR5*-edited cells (Fig. 5a). Even the threefold reduction of mRNA amounts in run #4, as compared to run #1, did not result in a substantial reduction of *CCR5* gene editing rate. On the contrary, *CCR2* off-target editing frequencies differed strongly between individual runs, particularly high *CCR2* editing rates of 18.5% were observed in run #3 (Fig. 5a). Together these findings confirm the pronounced impact of donor variability and dosage effects, which was also found in small-scale experiments with CCR5-Uco-TALEN and CCR5-Uco-hetTALEN in this (Fig. S2, S4) and previous work [24]. Notably, at increasing mRNA concentrations *CCR2* off-target editing frequency will still rise, when *CCR5* on-target editing already reached its maximum. However, donor variability also influences on-target to off-target ratio as seen in run #2. Therefore, mRNA amounts need to be decreased to stay in the lower linear range and thus avoid very high off-target rates. In fact, reduction of mRNA amounts in the fourth TCE run to one-third of the initial dose resulted in essentially conserved *CCR5* on-target activity with strongly reduced *CCR2* off-target editing (<2% in the final product).

### Detection of specific large deletions

Simultaneous cutting at both the *CCR5* on-target and the *CCR2* off-target sites might be repaired in different ways potentially leading to larger rearrangements, which are not detected by our GEF-dPCR described in the previous paragraph. To address this possibility, we designed various dPCRs (Fig. S5) and applied them for runs #2-#4 to the final products. The most likely outcome is the loss of the 15 kb fragment between the two TALEN binding sites at *CCR5* and *CCR2* and thus a double-knockout (dKO). As expected, based on off-target rates, the highest dKO rate (12.7%) was seen in run #3, while the numbers were much lower for runs #2 (7.4%) and #4 (2.2%). Interestingly, we occasionally also observed insertions of the cut-out 15 kb fragment into the *CCR5* on-target site, which obviously occurred at the second allele, albeit at low frequencies (1.1%, 1.3%, and 0.6%) for runs #2, #3 and #4, respectively (Fig. 5b). In contrast, no inversions of the 15 kb fragment were detected by sensitive dPCR.

### Biallelic *CCR5* editing

Generation of T cells resistant towards CCR5-tropic HIV requires knockout of both *CCR5* alleles. To quantify numbers of cells with mono- and biallelic *CCR5* knockout induced by CCR5-Uco-hetTALEN, we developed a dedicated technique based on single-cell high-resolution melting curve analysis (scHRMCA) and applied it to runs #2 and #4. The method is based on previous observations that small Indels in a PCR amplicon lead to detectable shifts in its melting temperature. In addition, presence of two different fragments results in formation of heteroduplexes, which changes the appearance of the melting curve [33–35]. By scHRMCA we observed more biallelic than monoallelic editing—about 67% of analysed cells for run #2 and 60% for run #4 showed biallelic melting curve profiles (Fig. 5c). Of all cells with biallelic editing, 56% (run #2) and 67% (run #4) showed homologous melting profiles, i.e. identical modifications of both alleles (Fig. 5c).

### CCR5-Uco-hetTALEN copy numbers

**Fig. 6:**
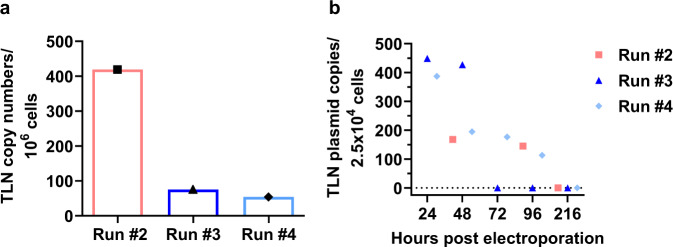
CCR5-Uco-hetTALEN mRNA and plasmid copy numbers determined by qPCR. **a** CCR5-Uco-hetTALEN copy numbers in RNA isolates for runs #2-#4 calculated based on the C_P_ values from a standard curve with defined TALEN plasmid copy numbers in final product. **b** CCR5-Uco-hetTALEN plasmid copy numbers in RNA isolates for runs #2-#4 calculated based on the C_P_ values from a standard curve with defined TALEN plasmid copy numbers in final product.

Exogenous nucleic acids might induce immunogenic reactions in patients, therefore high amounts of TALEN mRNA or plasmid DNA should be avoided in final product. We used real-time quantitative PCR to test for residual CCR5-Uco-hetTALEN mRNA and plasmid (possible contaminant from mRNA in-vitro production) in samples from runs #2 to #4 on day of harvest (216 h post electroporation). CCR5-Uco-hetTALEN copy numbers were calculated based on C_P_ values of standard curve and set in relation to 1 × 10^6^ cells. For run #2 419 CCR5-Uco-hetTALEN copies were detected in 1 × 10^6^ cells of the final product corresponding to 1 mRNA copy in app. 2400 cells; mRNA copy numbers were even lower for runs #3 (75 copies per 1 × 10^6^ cells, i.e. 1 copy/13,300 cells) and #4 (54 copies per 1 × 10^6^, i.e. 1 copy/18,500 cells) (Fig. 6a). In order to detect residual CCR5-Uco-hetTALEN plasmid, a specific qPCR assay was performed with RNA and gDNA isolates from different time points for runs #2-#4. Melting curves were recorded to ensure correct amplicons, as determined by plasmid control (mean melting temperature 89.18 ± 0.03 °C). No plasmid was detected in RNA (Fig. 6b; Fig. S6) or gDNA (Fig. S7) samples at 216 h post electroporation (final harvest) in any of the tested runs.

### Other potential off-targets in large-scale produced cells

**Fig. 7:**
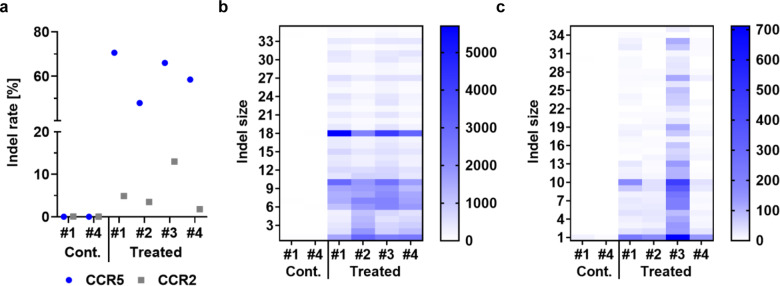
Amplicon NGS of samples from TCE processes. **a** Indel rates of *CCR5* (blue circle) and *CCR2* (grey square) from treated samples of runs #1-#4 and non-treated samples before electroporation from runs #1 and #3 (controls). **b** Numbers of *CCR5* Indel reads plotted against Indel sizes 1–35 for treated and non-treated samples (cont., before electroporation) from runs #1-#4. **c** Numbers of *CCR2* Indel reads plotted against Indel sizes 1–35 for treated and non-treated samples (cont., before electroporation) from run #1-#4.

To address overall off-target activity of CCR5-Uco-hetTALEN, in-silico analyses were performed using different online tools. Based thereon, we chose ten potential off-targets present in the TOP lists of different tools (*CCR2*, *CXCR6*, *GLP1R*, *CACNA1B*, *ASIC*, *SAMD12*, *ADYC2*, *PGC*, *MAT2B* and *UBXN10*). To investigate the actual off-target activity of our TALEN at these sites in the large-scale setting, we performed amplicon NGS for all four runs. As positive control, we also performed amplicon NGS at the on-target *CCR5*. Only samples from runs #1 and #3 taken before TALEN treatment served as negative controls, due to limited material and number of samples used for NGS analysis. Total Indel read counts for all potential off-targets but *CCR2* (*ADCY2*, *CACNA1B*, *CXCR6*, *MAT2B*, *ASIC*, *GLP1R* and *SAMD12*) showed no indication for CCR5-Uco-hetTALEN activity (Fig. S8, S9). Unfortunately, in all samples numbers of reads for potential off-targets PGC and UBXN10 were too low to allow conclusions (Fig. S8). Together and in line with previous small-scale experiments [25], *CCR2* was the only confirmed off-target with Indel rates between 1.8 and 13.0% in amplicon NGS data. On-target *CCR5* showed Indel rates of about 48–71% (Fig. 7a). Indel reads sorted by number of Indels showed a high prevalence of an 18 bp deletion at the *CCR5* locus (Fig. 7b) indicating microhomology-mediated end joining (MMEJ) as a major repair pathway. For *CCR2* we found preferential occurrence of 1 bp, 9 bp and 10 bp deletions. Overall, the majority of deletions were ≤15 bp (Fig. 7c).

### Proliferation capacity and cytokine secretion of large-scale produced cells

**Fig. 8:**
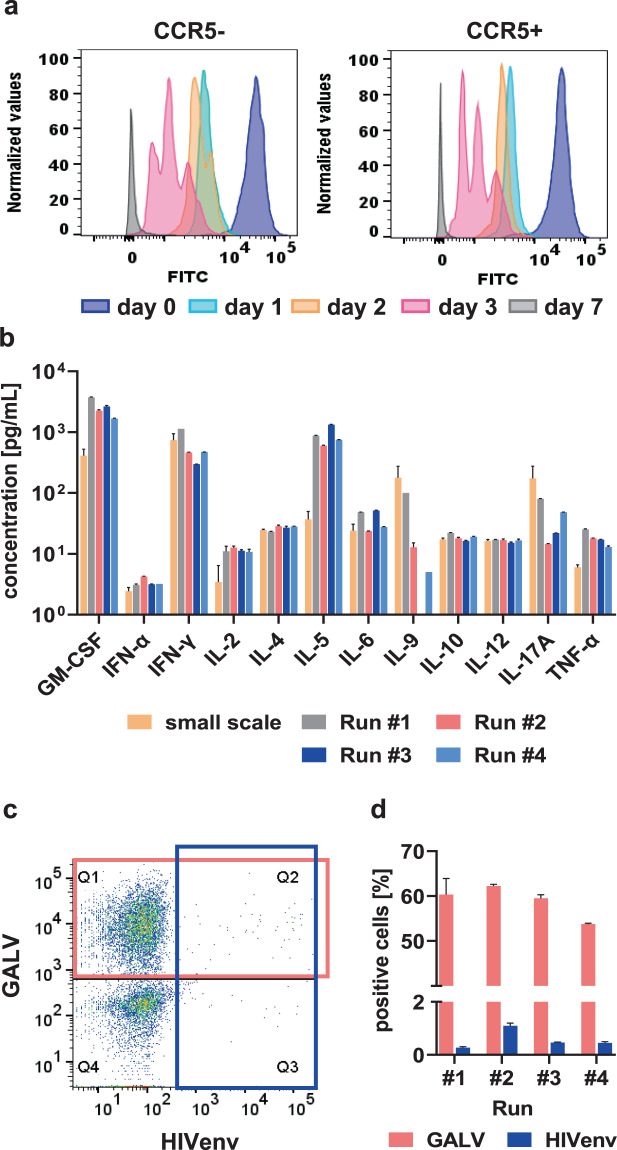
Proliferation capacity, cytokine secretion and viral susceptibility of TCE-produced *CCR5*-edited cells. **a** Monitoring of cell proliferation based on dilution of CellTrace CSFE dye over 7 days of measurement for run #1. Cells were stained for CCR5 expression and differences in proliferation were measured on days 0, 1, 2, 3 and 7 after staining with CellTrace CSFE. Exemplary graph from cells of run #1. **b** Cytokine secretion in medium harvested from restimulated TCE-produced CCR5-edited cells in comparison with medium from non-edited small-scale control cells (small scale). Each measurement was done in technical duplicates, small-scale values represent mean values for 3 different donors. Error bars indicate s.d. **c** Exemplary dot plot for the gating strategy to examine susceptibility of large-scale produced cells towards transduction with GALVenv- and HIVenv-pseudotyped lentiviral vectors with cells from run #2. **d** Flow cytometry results for the simultaneous transduction of large-scale produced CCR5-edited cells from runs #1-#4 with GALVenv- and HIVenv-pseudotyped lentiviral vectors. Transductions were performed in triplicates for each run. Error bars represent s.d.

The production process and expansion for 12 days might have influenced the T cells’ capacity to proliferate. Therefore, we assessed the proliferation potential of thawed large-scale produced CCR5-edited CD4^+^-T cells from runs #1-#4 after restimulation with anti-CD3/anti-CD28 conjugated beads by staining with CellTrace CFSE and flow cytometry. Proliferation was measured over a time period of 7 days after restimulation and analysed separately for CCR5-positive and CCR5-negative cells. We observed no substantial differences in cell-division rates of CCR5-positive or CCR5-negative cells (Fig. 8a, S10). Notably, *CCR5*-Indel rates remained stable 9 days after restimulation supporting identical proliferation capacity of CCR5 WT and knockout cells (data not shown). Moreover, even 9 days post restimulation thawed large-scale produced cells showed no exhaustion phenotype (data not shown). To further examine any impairment of large-scale produced T cells after *CCR5* editing, the secretion of specific cytokines (GM-CSF, IFN-α, IFN-γ, IL-2, IL-4, IL-5, IL-6, IL-9, IL-10, IL-12p70, IL-17A and TNF-α) into the medium was measured on day 9 after restimulation of cells. Large-scale produced *CCR5*-edited cells showed a similar cytokine secretion profile as small-scale, non-edited control cells from day 12 after first stimulation (Fig. 8b).

### HIV susceptibility of large-scale produced cells

In order to test for functional resistance towards an infection with HIV, large-scale produced *CCR5*-edited CD4^+^-T cells from all runs were simultaneously transduced with GALVenv- and HIVenv-pseudotyped lentiviral vectors and analysed by flow cytometry [13]. While transduction with GALVenv-pseudotyped lentiviral vectors resulted in 53.8–62.2% transgene-positive cells, transduction rates of HIVenv-pseudotyped lentiviral vectors remained very low between 0.3 and 1.1% (Fig. 8c, d). In comparison, small-scale experiments with mock control CD4^+^-T cells showed substantially higher transduction rates with HIVenv-pseudotyped lentiviral vectors (12.7–26.3%) at identical multiplicity of infection.

## Discussion

Since the introduction of ART, HIV infection has become a chronic but treatable disease. Nevertheless, lifelong therapies lead to a variety of disadvantages including drug resistances or interactions in an aging population. In addition, HIV infections remain a challenge in other newly developed therapies, like immunotherapies (e.g. CAR T cells). Therefore, permanent protection from HIV by the means of genome editing has been suggested as a promising therapeutic approach to overcome current limitations of ART [36]. Two general approaches based on ex-vivo cell modification have been tested in clinical settings—modification of HSC and protection of T lymphocytes (clinicaltrials.gov NCT01734850 [1],). Both approaches have certain advantages and shortcomings. Indeed, efficient high-level protection of HSC would potentially ensure lifelong production of HIV-resistant cells, including not only CD4^+^-T cells, but also other HIV targets, namely macrophages. This has been considered a sine qua non for functional HIV cure as seen in the Berlin patient [6, 37]. However, in contrast to the allogeneic setting of the Berlin patient, modification of autologous HSC would only protect de-novo generated T cells. All CD4^+^-T cells already present in the patient would potentially become HIV targets, which might create new HIV reservoirs [37] and impends the loss of large parts of the acquired adaptive immunity thus conferring high infection risks, which is particularly problematic for HIV patients. Moreover, T cells generated de-novo in the presence of HIV antigens could be expected to be tolerized against the virus. In contrast, protection of existing T cells against HIV would perpetuate pre-existing immune answers of HIV patients, including those against HIV. In fact, including HIV-reactive and, at the same time, HIV-resistant T cells has been suggested an indispensable element of HIV cure approaches [36–39]. Importantly, genetic protection, e.g. based on *CCR5* knockout as shown in this work, could readily be combined with further modifications, such as introduction of HIV-directed chimaeric antigen receptors (CARs). Finally, T lymphocytes are largely resistant to malignant transformation [40]—a fact also underlined by the large numbers of gene-modified T cells infused in thousands of patients without severe side effects. On the downside, only limited proportions of T cells are long-lived, although gene-modified T cells were detected for more than 10 years even in the very early studies [41]. Altogether, combined approaches protecting both HSC and (armoured) T cells might be the ultimate solution.

We previously developed a TALE nuclease (CCR5-Uco-TALEN) that targets *CCR5*, which is subsequently being genetically knocked out by cellular repair mechanisms (NHEJ and MMEJ) [23, 24]. Recently, we have designed and tested an optimised, obligatory heterodimeric version (CCR5-Uco-hetTALEN) with strongly reduced off-target activity [25].

Translation of genome-editing concepts towards clinical application is still challenging. This not only includes design and organisation of clinical studies, but also large-scale manufacture of the (cellular) Gene Therapy Medicinal Products for human application in accordance with the regulations applying for GMP and biosafety, i.e. in dedicated clean rooms by highly trained staff. The biosafety issue becomes particularly important, when it comes to CD4^+^-T cells from HIV-positive patients, which can only be handled under additional biosafety measures.

Here we present the development of a GMP-compatible protocol at the CliniMACS Prodigy for automated production of *CCR5*-edited CD4^+^-T cells in a closed system, which could overcome some of the above challenges. Automated manufacturing of cell products reduces the probability of human errors. In addition, the closed system decreases risks of contamination of cell products as well as the need of extended decontamination between individual production processes. Over the last years several protocols for the automated separation and transduction of T cells and CD34^+^ cells have successfully been established at the CliniMACS Prodigy [42–47]. We here add a new process combining targeted genome editing based on mRNA transfection of a TALE nuclease and CD4^+^ cell expansion to generate large numbers of *CCR5*-edited CD4^+^-T cells.

We performed four runs at the CliniMACS Prodigy using an adapted protocol for gene editing of T cells based on mRNA electroporation including (i) separation of CD4^+^ cells from buffy coats of healthy donors and activation of T cells, (ii) electroporation with (TALEN-) mRNA, (iii) expansion of T cells and (iv) harvest of the cell product. Separation of CD4^+^ cells resulted in high numbers of CD4^+^-T cells with minor co-enrichment of monocytes, obviously due to low CD4 expression in a fraction of those cells [48, 49]. Over the whole run T cells expanded at least 11-fold reaching cell numbers between 1.65 and 5.00 × 10^9^, except for run #2 with only 1 × 10^8^ cells in final product. Impaired expansion in run #2 was obviously due to a failure in the centralised CO_2_ supply of our research building that unfortunately occurred during the T-cell activation phase of that run. Final cell numbers obtained with our protocol were similar to those reported by other groups, even though fold-expansions were comparatively lower. However, in those other studies, the CliniMACS Prodigy was used to carry out T-cell transduction [50, 51]. In contrast, our process included electroporation of cells, introduction of a TALE nuclease and 24 h culture at 32 °C. As evident from Fig. 3a, in conjunction these three manipulations resulted in strong, but transient growth delay, importantly without negative impact on the subsequent expansion capability. Importantly, cells number of 1–5 × 10^9^ would be sufficient for a clinical study with doses of 1–5 × 10^7^ cells/kg. App 1 × 10^10^ cells per patient were used in the landmark study by Tebas et al [1]. In that study, efficacy (in terms of complete HIV suppression during ART interruption) was seen for one patient heterozygous for the Δ32 allele. In that patient, ZFN-mediated Indels were detected in app. 20% of the *CCR5* alleles. Since those Indels could be present in both the WT and the Δ32 allele, the observed clinical efficacy was apparently mediated by a total of app. 10^9^ *CCR5* k.o. CD4^+^ cells. In our production process we observed biallelic editing, i.e. complete knockout, in up to 50% of the final product, which corresponds to app. 0.8 to up to 2.5 × 10^9^ protected CD4^+^ cells. In view of the published results [1], already these numbers might be sufficient for at least transient HIV suppression. Notably, higher cell numbers could be obtained with an additional expansion step in a chamber of higher volume. A significant finding was the high percentage (25–42%) of long-lived central memory T cells in the final products, similar to previous results [52]. In two runs (#2 and #4), we also observed 5.0% and 1.9% stem cell memory T cells, respectively. However, these rates were much lower rate than reported by Mock et al [53]. Moreover, we did not observe exhaustion of edited T cells, neither after the 12-day production process nor after restimulation. Only a small fraction of cells from run #2 showed low rates of PD-1 and TIM3-positive cells on day 12 of the process, but failure of the CO_2_ supply during this run might have affected the cells. No elevated expression of PD-1 or TIM3 was observed on day 9 after restimulation in those cells. In addition, the cytokine secretion of restimulated large-scale produced cells showed no impairment of *CCR5*-edited cells, as already shown in small-scale experiments [25].

Crucially, we observed high *CCR5* gene editing rates of 65% and above in all four runs. Except for run #3, all runs showed low Indel rates at off-target *CCR2* (≤6.5%). As seen for our previously performed small-scale experiments, simultaneous editing at on-target *CCR5* and off-target *CCR2* might also lead to the deletion or insertion of a 15 kb fragment between the two TALEN binding sites at these loci. However, except for run #3 the numbers of deletion (dKO) or insertion (Ins) remained low in the large-scale produced cells. Using a new single-cell HRMCA assay we empirically confirmed high proportions of T cells with biallelic knockout, a sine qua non for future clinical studies. Even though we are not able to rule out wrong calling of some of the tested cells, e.g. due to large deletions found in one allele, we expect this number to be small. Our NGS data at the on-target locus *CCR5* showed that most edited sequences had Indels <30 bp, which are detectable in our single-cell HRMCA. Besides, the number of detected large deletions was low in the two tested runs—below 8% in run #2 and at around 2% in run #4 in the two tested runs. Consequently, for both tested runs the majority of edited Indels were biallelic. Statistically, with these high *CCR5* Indel rates biallelic gene editing at the on-target locus could be expected in at least 40% of the cells (65% *CCR5-*edited cells of which 65% are biallelic).

The monitoring of CCR5 expression during the process for runs #2-#4 revealed a decrease in CCR5-positive cells between days 3–5 to day 12 of the process. The strongest decrease in CCR5 expression was seen for run #3, which also showed the highest rates of biallelic *CCR5* editing. The reduction of CCR5 expression also translated into low transduction rates with HIVenv-pseudotyped lentiviral vectors (0.3–1.1% positive cells) in edited cells. Obviously, since the expression of CCR5 in human T cells highly depends on the individual donor, the activation status and the subtype of T cells, a matched mock control would be necessary to assess the actual percentages of CCR5 reduction and its impact for each run, but such controls were not available for large-scale runs. However, we could show a clear correlation between measured *CCR5* editing, CCR5 surface expression and susceptibility to HIVenv- pseudotyped lentiviral vectors for cells from the same donors in parallel small-scale studies [25].

The data obtained in this work also confirmed remarkable differences in *CCR5* on-target as well as *CCR2* off-target activities for individual donors, which were also seen in small-scale experiments [25]. Even with the same mRNA amount used for the same cell concentration during electroporation Indel rates differ up to twofold [24, 25]. At too-high TALEN doses, the on-target activity reaches a plateau, whereas the off-target cutting still increases linearly. Moreover, as seen during run #3 extensive cutting at the off-target site also leads to higher numbers of larger deletions thus increasing the number of cells with *CCR5*, but also *CCR2* knockout. Hence, mRNA concentrations within this plateau of *CCR5* editing might lead to elevated off-target activity in some donors. In this context, it is particularly relevant to note that up-scaling of mRNA amounts in correlation with cell numbers from small-scale to large-scale experiments requires careful attention. Indeed, in the final run we used three times less mRNA without impairing on-target efficiency, but a strong reduction in off-target activity, which was in some contrast to our small-scale experiments during titration of the mRNA. This observation might be due to differences in cuvettes used for small- and large- (CliniMACS Prodigy) scale, but also the general optimisation of the GMP process. When setting up a clinical production process, examination of the right dosing is crucial.

Notably, as already indicated by the small-scale experiments [25], amplicon NGS analysis of 10 potential, in-silico predicted off-targets in large-scale produced cells revealed no evidence for CCR5-Uco-hetTALEN activity at any off-target site but *CCR2*. Also, analysis of residual CCR5-Uco-hetTALEN plasmid and mRNA by qPCR showed rapid decay of exogenous nucleic acids. Indeed, we detected only single mRNA copies per >10,000 cells in final products of runs #3 and #4. Even for run #2, where cell growth was impaired for technical reasons, mRNA copy numbers were below 1 in 2000 cells. No plasmid DNA was found in the final product for any of the runs.

In summary, we established a GMP-compatible, automated protocol to produce gene-edited CD4^+^-T cells based on TALEN mRNA-electroporation. Using this protocol in conjunction with our newly optimised CCR5-Uco-hetTALEN, we were able to obtain clinically relevant numbers of *CCR5*-edited primary CD4^+^-T cells in just 12 days. Thus, the established protocol lays the base for translating novel cell therapies with (largely) protected T lymphocytes towards clinical application in HIV-positive patients. Based on the flexibility of the CliniMACS platform, the process presented here can readily be combined with further modifications, for example CAR transduction. HIV-protected CAR T cells have been suggested as part of future HIV cure approaches [38], but could already today be extremely useful for HIV-positive patients suffering from malignancies that could be treated with CAR T cells [54]. Furthermore, knockout of *CCR5* might be combined with nucleases that cut out the HI-provirus in infected cells, like Brec1 [21] or with strategies to reactivate and eliminate the viral reservoir.

## Supplementary information

Supplementary information
