# Supplementary material for: Automated production of CCR5-negative CD4+-T cells in a GMP-compatible, clinical scale for treatment of HIV-positive patients
Source: Gene Ther. 2021 Apr 19;28(9):572–87. doi: 10.1038/s41434-021-00259-5 (PMC8455337; doi:10.1038/s41434-021-00259-5)
Supplement: Supplementary file 1 — Supplementary information [file 41434_2021_259_MOESM1_ESM.pdf]

Automated production of CCR5-negative CD4^{+}-T cells in a GMP-compatible, clinical scale for treatment of HIV-positive patients (Supplementary data)

Schwarze et al.: Supplementary Tables

Table S1: Selected loci of heterodimeric CCR5-Uco-hetTALEN for off-target analysis

(closest) gene	mismatches	chromosome	spacer length	loci
CCR5	0/0	3	18	exon
CCR2	3/3	3	18	exon
CXCR6	7/5	3	12	exon
GLP1R	5/5	6	24	exon
CACNA1B	7/6	9	28	intron
ASIC-202	5/8	17	24	intron
SAMD12	6/4	8	22	intron
ADCY2	5/4	5	15	intron
PGC	7/4	6	17	promotor
MAT2B	7/3	5	19	intergenic
UBXN10	7/3	1	24	intergenic

Table S2: Primer and probes used

Name	Sequence	Purification	Modification
CCR5 fw	CTGCCTCCGCTCTACTCACT	HPSF	
CCR5 rv	CCCAGAAGGGGACAGTAAGA	HPSF	
CCR5 ref	CTTTGGTTTTGTGGGCAACATGCT	HPLC	5‘ HEX 3‘-BHQ1
CCR5 mut	CTGCAAAAGGCTGAAGAGCATGAC	HPLC	5‘ FAM 3‘-BHQ1
CCR2 fw	CAAATTGGGGCCCAACTC	HPSF	
CCR2 rv	GCCCACAATGGGAGAGTAATA	HPSF	
CCR2 mut	CTGCAAAAAGCTGAAGTGCTTGACTG	HPLC	5‘ FAM 3‘-BHQ1
hEPOR fw	GCTGCCAGCTTTGAGTACACTA	HPSF	
hEPOR rv	GAGATGCCAGAGTCAGATACCACAA	HPSF	
hEPOR ref	TTCTGAGGCGCCACTTTTGCAAGACC	HPLC	5‘ FAM 3‘-BHQ1
Inv1fw	GAAGCAAATCGCAGCCC	HPSF	
Int1rv	TATGCACAGGGTGGAACAAG	HPSF	
hetTALENfw	CAAGCACCTCGGAGGATCAC	Salt-free	
hetTALENrv	ATAGGCAGATTGTAGCCGCC	Salt-free	
IPO8fw	GACCGTTCCTCCTGAGACTC	Salt-free	
IPO8rv	TCCTGGGCTTCCATATCGTTC	Salt-free	
nesPCRfw	CATTCATGGAGGGCAACTAAATAC	HPSF	
nesPCRrv	CGATTGTCAGGAGGATGATG	HPSF	
HRMfw	GGTTTTGTGGGCAACATGCTGG	HPSF	
HRMrv	GGTCAGAGATGGCCAGGTTG	HPSF	

Schwarze et al.: Supplementary Figures

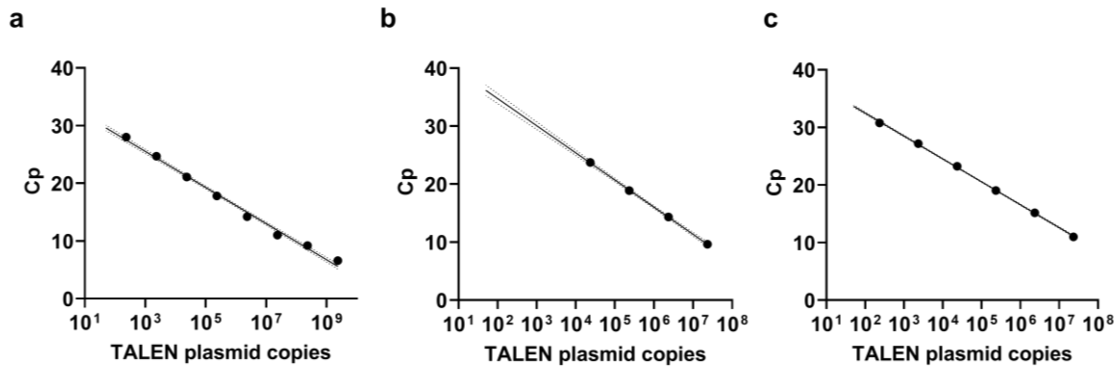

Figure S1: Standard curves for dilution of CCR5-Uco-hetTALEN plasmid for detection of CCR5-Uco-hetTALEN mRNA and plasmid. CCR5-Uco-hetTALEN plasmids L+R were diluted resulting in indicated copy numbers. C_q values were determined by qPCR in triplicates. A line (semilog) was fitted to data points by nonlinear regression with least square regression using GraphPad Prism 8.4.3. Dotted lines indicate prediction bands with a confidence interval of 95%. (a) qPCR for detection of CCR5-Uco-hetTALEN with primers hetTALENfw and hetTALENrv. R^2 value = 0.9921. (b) qPCR for detection of CCR5-Uco-hetTALEN plasmid in RNA isolates with primers Kanfw and Kanrv. R^2 value = 0.9959. (b) qPCR for detection of CCR5-Uco-hetTALEN plasmid in RNA isolates with primers Kanfw and Kanrv. R^2 value = 0.9993.

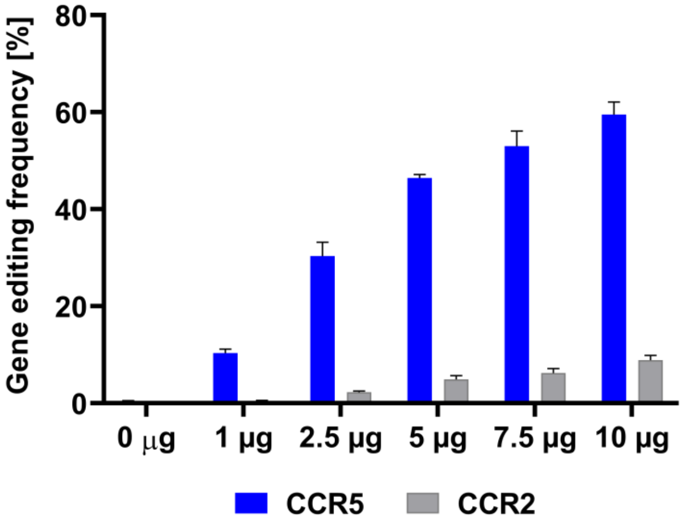

Figure S2: Titration of CCR5-Uco-hetTALEN L+R mRNA purchased from BioNTech in small-scale experiment. $2x10^6$ human $CD4^+$ T cells separated from freshly isolated PBMCs of one buffy coat (obtained from the Transfusion Medicine at the UKE after informed consent of healthy blood donors) were electroporated with indicated amounts of CCR5-Uco-hetTALEN L+R mRNA per arm. *CCR5* and *CCR2* gene editing rates were determined by GEF-dPCR. View details of electroporation in Schwarze et al. (1). n=3. Error bars show s.d..

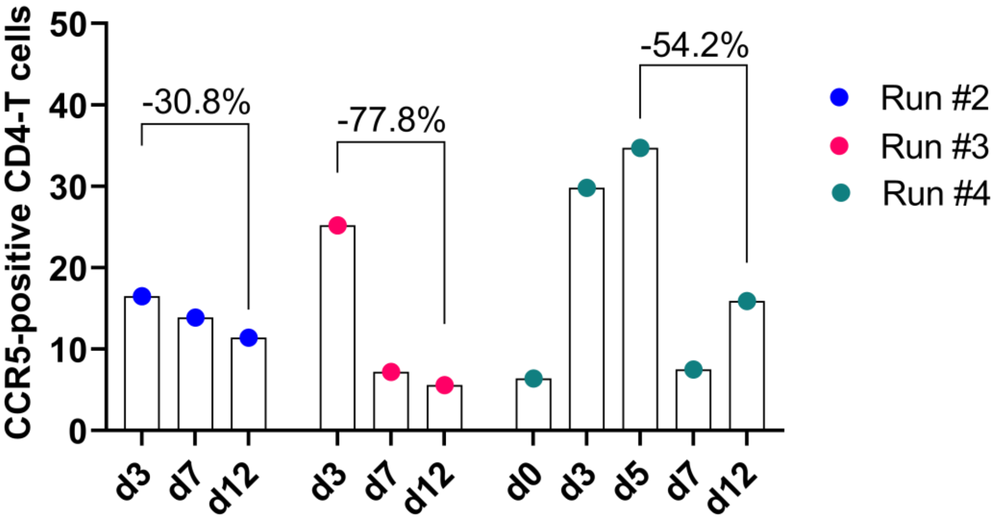

Figure S3: TCE-produced *CCR5*-edited cells show reduced CCR5 expression. Summarised data on CCR5 expression measured on different days during the TCE process for runs #2-#4.

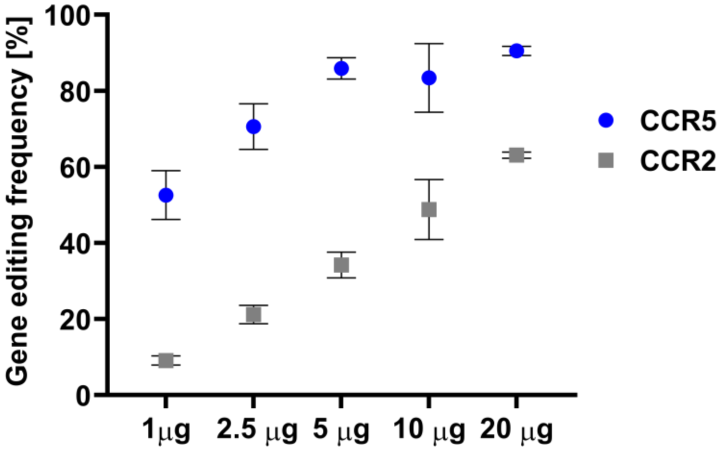

Figure S4: Titration of homodimeric CCR5-Uco-TALEN L+R mRNA in small-scale experiments. $1.5x10^6$ PM1 cells (2) were electroporated with indicated amounts of TALEN mRNA per arm using the following electroporation parameter at the Gene Pulser X cell Electroporation system: 350 Volt, 3 square-wave-pulse for 5 ms each with 0.1 ms pause between pulses. Electroporation protocol and culture of PM1 were performed as described for Jurkat cells (1). *CCR5* and *CCR2* gene editing rates were determined by GEF-dPCR. n=3. Error bars show s.d..

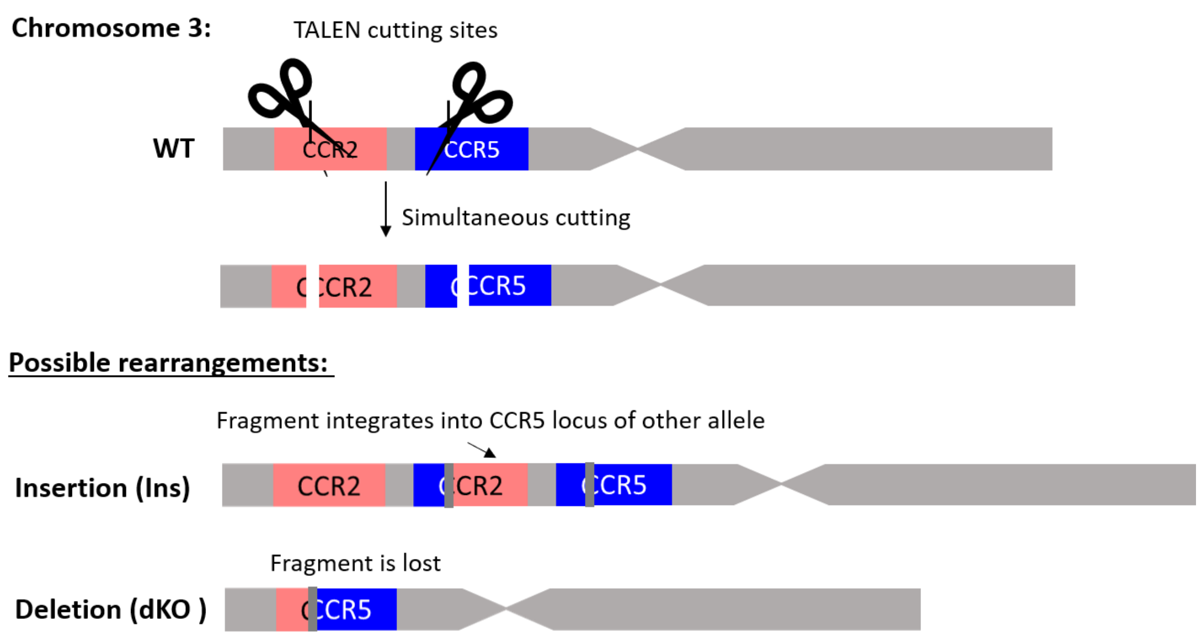

Figure S5: Possible chromosomal rearrangements after CCR5-Uco-hetTALEN L+R mRNA treatment. Schematic diagram of chromosome 3 with location of CCR5-Uco-hetTALEN binding sites at loci *CCR2* and *CCR5*. Most probable chromosomal rearrangements due to simultaneous cutting at both loci and excision of a 15-kb fragment: (Ins) Insertion of the 15-kb fragment into the *CCR5* locus at the other allele. (dKO) Removal of the whole 15-kb fragment and re-ligation of chromosome. Dark grey bars indicate possible Indels at repaired sites.

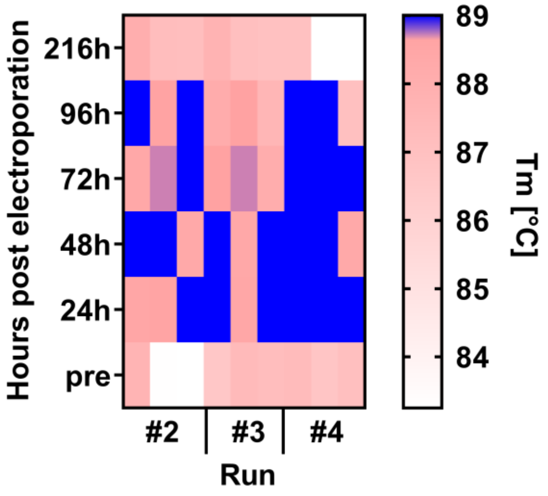

Figure S6: Detection of residual CCR5-Uco-hetTALEN production plasmids in RNA isolates from CCR5-Uco-hetTALEN L+R mRNA treated cells. All samples (triplicates) from different time points of runs #2-#4 were tested by qPCR. Heatmap shows melting temperature (Tm in °C) of amplified products. Blue colour indicates correct amplicon with a melting temperature between 89.0-89.3°C, as derived from positive control (diluted CCR5-Uco-hetTALEN plasmid). Lower melting temperatures are indicated in colours from pink to white.

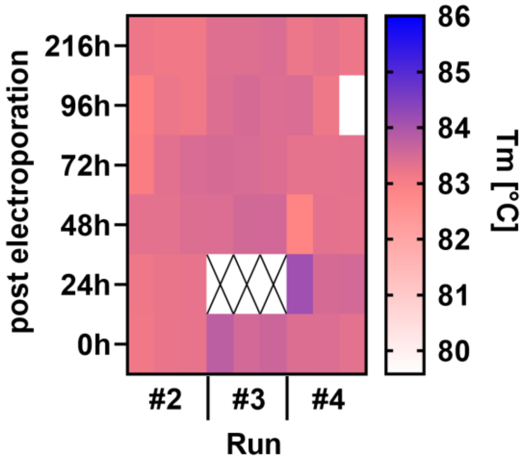

Figure S7: Detection of residual CCR5-Uco-hetTALEN production plasmids in gDNA isolates from CCR5-Uco-hetTALEN L+R mRNA treated cells. All samples (triplicates) from different time points of runs #2-#4 were tested by qPCR. Heatmap shows melting temperature (Tm in °C) of amplified products. Blue colour indicates correct amplicon with a melting temperature between 85.0-85.9°C, as derived from positive control (diluted CCR5-Uco-hetTALEN L+R plasmid). Lower melting temperatures are indicated in colours from pink to white. Cells with no measurement are marked with X.

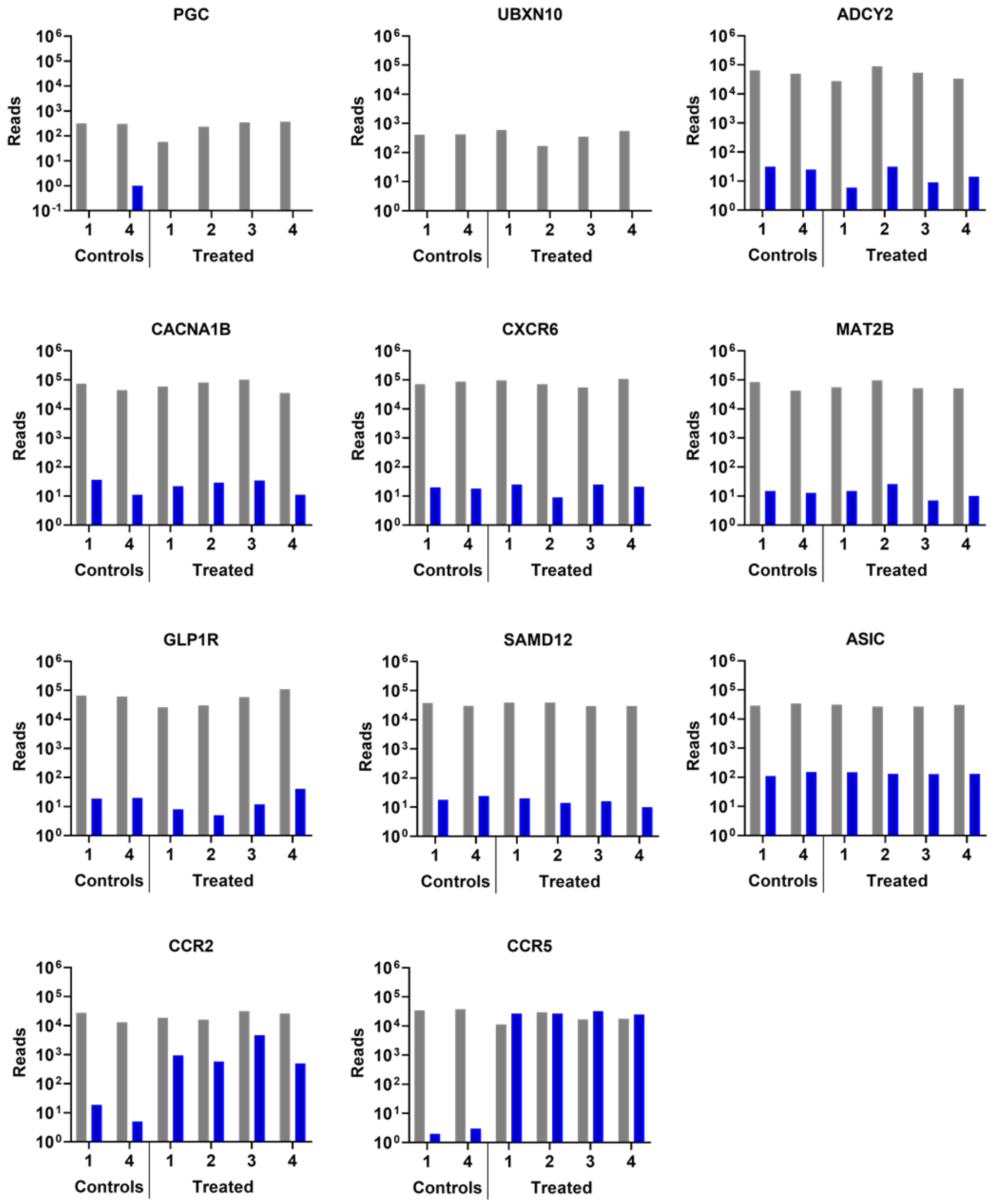

Figure S8: Next-generation amplicon sequencing data (read counts) from TCE runs #1-4 (216 h post electroporation) treated with CCR5-Uco-hetTALEN L+R mRNA and 2 control samples pre electroporation from runs #1 and #4 for on-target *CCR5* and 10 potential off-targets. Reads containing insertions or deletions (Indels) at the TALEN-binding sites were counted as Indel reads (right, blue bars), whereas all other reads were considered non-edited (left, grey bars).

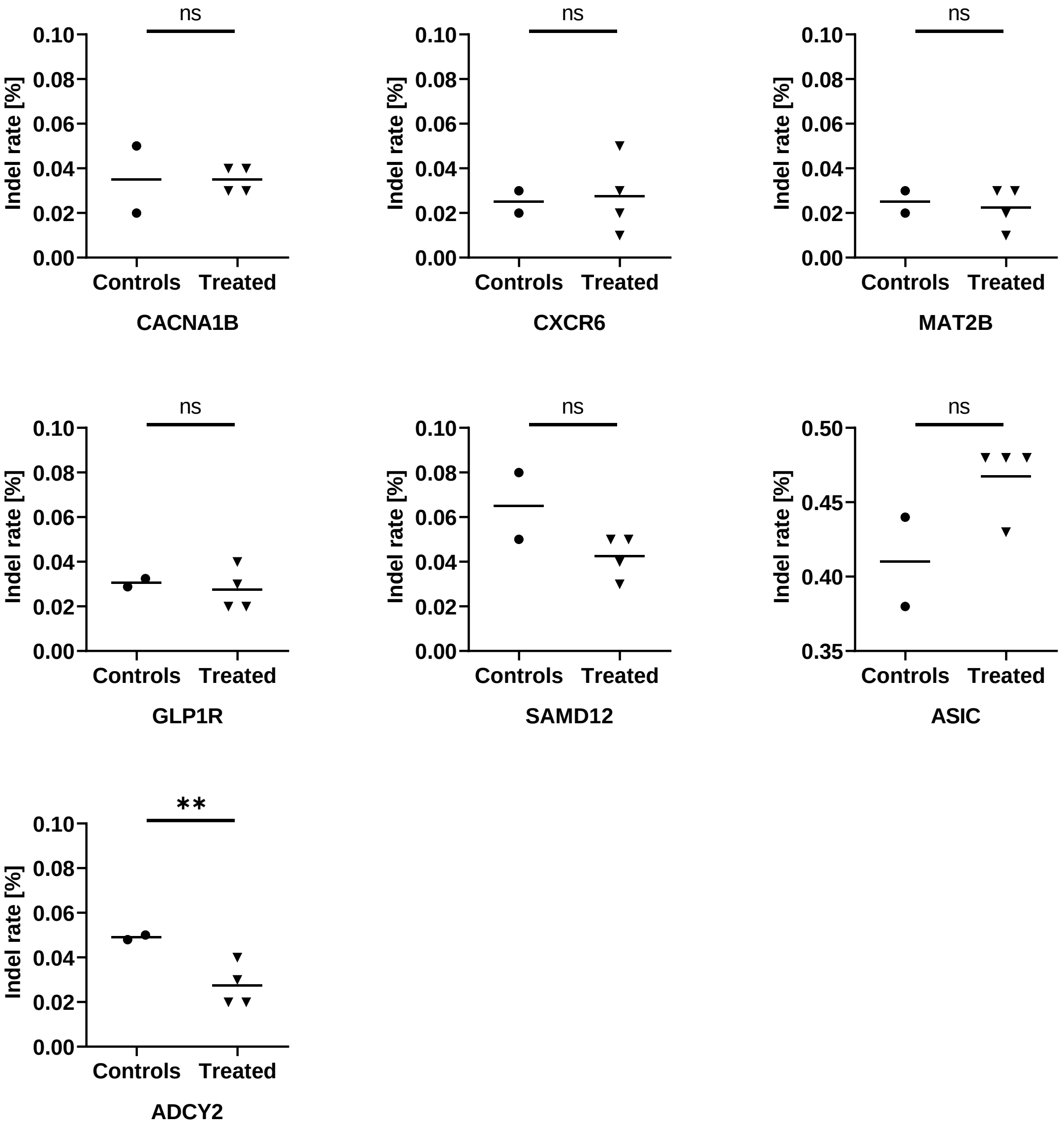

Figure S9: Next-generation amplicon sequencing data (read counts) from TCE runs #1-#4 (216 h post electroporation) treated with CCR5-Uco-hetTALEN L+R mRNA and 2 control samples pre electroporation from runs #1 and #4 for 7 potential off-targets: *CACNA1B*, *CXCR6*, *MAT2B*, *GLP1R*, *SAMD12*, *ASIC, ADYC2*. Reads containing insertions or deletions (Indels) at the TALEN-binding sites were counted as Indel reads, whereas all other reads were considered non-edited. Indel rates were calculated using the ratio of reads containing Indels to all reads. Statistical analysis of Indel ratio was done using a one-tailed Welch's t test with a confidence interval of 95%. P values: ns $p > 0.1234$, ** $p > 0.002$

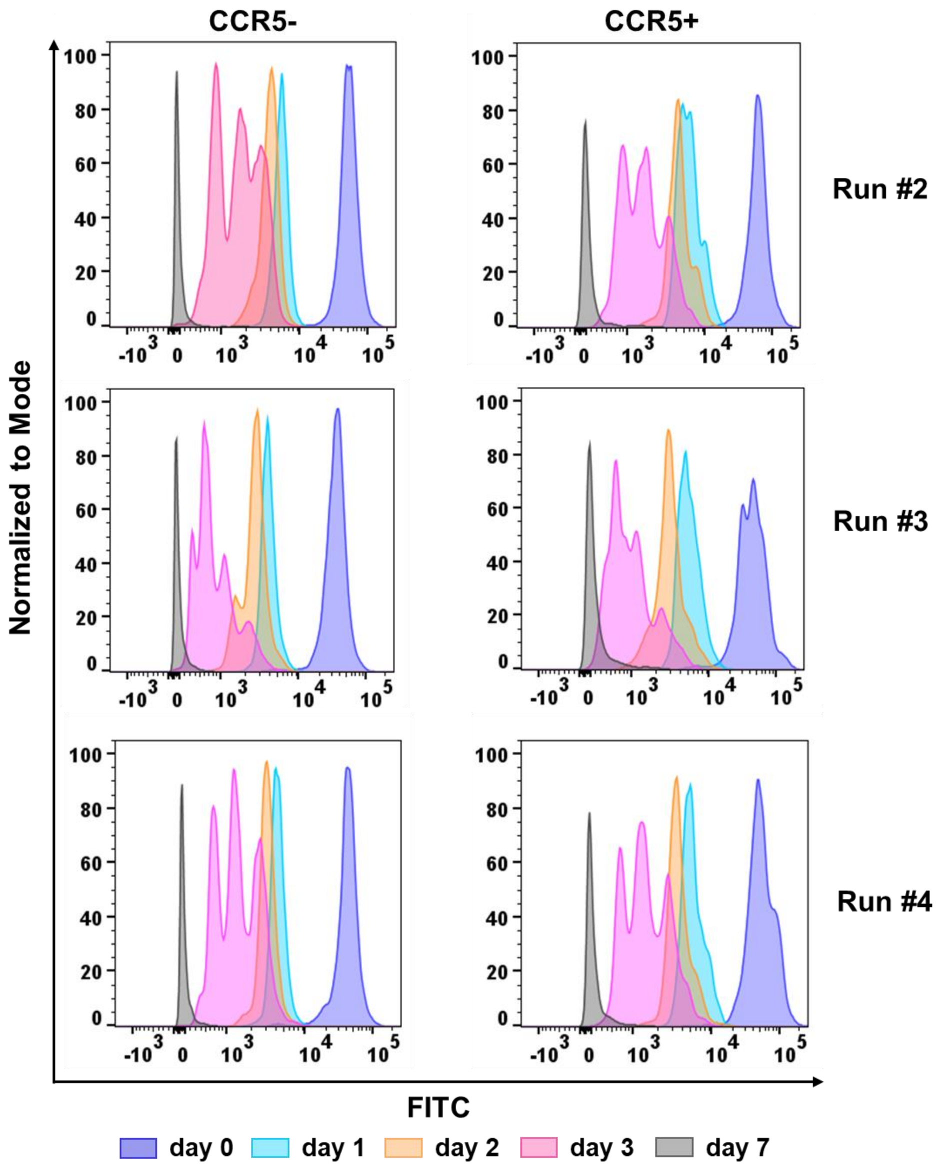

Figure S10: Proliferation of TCE produced *CCR5*-edited cells. Monitoring of cell proliferation based on dilution of CellTrace CSFE dye over 7 days of measurement for runs #2-#4. Cells were stained for CCR5 expression, and differences in proliferation were measured on days 0, 1, 2, 3 and 7 after staining with CellTrace CSFE.

References

1. Schwarze LI, Głów D, Sonntag T, Uhde A, Fehse B. Optimisation of a TALE nuclease targeting the HIV coreceptor CCR5 for clinical application. Gene Ther. :contemporaneously submitted.

2. Lusso P, Cocchi F, Balotta C, Markham PD, Louie A, Farci P, et al. Growth of macrophage-tropic and primary human immunodeficiency virus type 1 (HIV-1) isolates in a unique CD4+ T-cell clone (PM1): failure to downregulate CD4 and to interfere with cell-line-tropic HIV-1. J Virol. 1995;69(6):3712–20.
